# Phase Interrogation Used for a Wireless Passive Pressure Sensor in an 800 °C High-Temperature Environment

**DOI:** 10.3390/s150202548

**Published:** 2015-01-23

**Authors:** Huixin Zhang, Yingping Hong, Ting Liang, Hairui Zhang, Qiulin Tan, Chenyang Xue, Jun Liu, Wendong Zhang, Jijun Xiong

**Affiliations:** 1 Key Laboratory of Instrumentation Science & Dynamic Measurement, Ministry of Education, North University of China, Taiyuan 030051, China; E-Mails: zhanghx@nuc.edu.cn (H.Z.); hongyingping_2014@163.com (Y.H.); liangtingnuc@126.com (T.L.); zhanghairuinuc@126.com (H.Z.); tanqiulinnuc@126.com (Q.T.); xuechenyangnuc@126.com (C.X.); liujunnuc2014@126.com (J.L.); zhangwendongnuc@126.com (W.Z.); 2 Science and Technology on Electronic Test & Measurement Laboratory, North University of China, Taiyuan 030051, China

**Keywords:** LC resonant sensor, resonant frequency, high temperature, pressure measurement

## Abstract

A wireless passive pressure measurement system for an 800 °C high-temperature environment is proposed and the impedance variation caused by the mutual coupling between a read antenna and a LC resonant sensor is analyzed. The system consists of a ceramic-based LC resonant sensor, a readout device for impedance phase interrogation, heat insulating material, and a composite temperature-pressure test platform. Performances of the pressure sensor are measured by the measurement system sufficiently, including pressure sensitivity at room temperature, zero drift from room temperature to 800 °C, and the pressure sensitivity under the 800 °C high temperature environment. The results show that the linearity of sensor is 0.93%, the repeatability is 6.6%, the hysteretic error is 1.67%, and the sensor sensitivity is 374 KHz/bar. The proposed measurement system, with high engineering value, demonstrates good pressure sensing performance in a high temperature environment.

## 1. Introduction

There is much innovative research on wireless passive LC resonant sensors for special situations where traditional wired sensors could not be applied. For example, measurements of human intraocular pressure [1,2], temperature in harsh environments [3–5], quantifying packaged food quality [6,7], humidity [8,9], and so on. The Georgia Institute of Technology has designed a wireless high temperature pressure sensor using a low temperature co-fired ceramic (LTCC) material. However, the sensor was only tested to 450 °C [10,11]. Ceramic-based passive LC wireless pressure sensors are applied in ultra- high temperature and other harsh environments for pressure, temperature and other parameter measurements, since the ceramic material has excellent mechanical stability in such high-temperature environments. Xiong *et al.* made numerous efforts in designing sensitive heads and have already achieved great results in high temperature and pressure monitoring processes. For example, a high temperature and pressure sensitive element made of high temperature co-fired ceramic (HTCC) with a sensitivity of 860 Hz/bar and operation temperature at 600 °C [12], or made of low temperature co-firing ceramic (LTCC) with a sensitivity of 31 KHz/bar at 600 °C [13]. However, these experiments were realized by an impedance analyzer, including the pressure performance of sensors at room temperature and the temperature-dependent resonant frequency drift with atmospheric pressure under a high temperature environment, which is not enough to obtain the characterization of resonant sensors under high-temperature environment conditions.

Some researchers have reported front-end circuit concepts for wireless sensor readout systems. These read circuits are designed by demodulating the changes of impedance parameters of the read antenna terminal due to the mutual coupling. Nopper *et al.* extracted the resonance frequency signal of a sensor at a distance by multiplying the source signal of the read antenna terminal and the modulation signal produced by the mutual coupling, then filtering and demodulating the real part of the impedance of the read antenna terminal [14], however, the test accuracy of the read circuit was strongly influenced by the *Q* factor of the remote sensor. Coosemans *et al.* [15] studied the read circuit based on a voltage controlled oscillator (VCO), which generated a frequency selecting circuit to drive the read antenna coil. The VCO output signal would produce a mutation point at the resonant frequency of the remote sensor in a sweep period. However, the results were not very satisfactory. Salpavaara *et al.* [16] studied the hardware detection circuit of phase differences, which compensated the resonant frequency changes with coupling distance, and built a system for pressure garment monitoring. Bao *et al.* [17] built a phase difference detection method based on DSP, and amplified the weak signal over a long distance through differential mode, however, the working frequency band is very low, less than 1 MHz. The sensitive components and read circuits in the mutual coupling systems mentioned above were designed to be applied in a more secure environment. In other words, when the sensors operated, only the sensitive capacitance of the sensor would change, but other parameters of the sensor would stay the same. For example, pressure measurement of food packages, humidity, pressure garments, intraocular pressure (IOP) monitoring systems, and so on. Sardini and Serpelloni *et al.* [18] have presented research on conditioning electronics for temperature monitoring by extracting the real component and phase of the impedance. They developed a sensor designed and fabricated with hybrid MEMS technology which is positioned in a high temperature airtight environment, while the readout antenna is outside in a safe zone. The measurement temperature of their sensors can reach as high as 330 °C.

In this paper, we present a complete wireless passive measurement system of temperatures up to 800 °C inside a hermetic pressure and high temperature environment. This system comprises a LC resonance sensor based on alumina ceramic material made by HTCC technology, a read antenna, a RF connection cable, a thermal insulation structure, a resonant phase-shift reading unit, and a computer to monitor and process data. In addition, we theoretically analyze the impedance parameters of the wireless passive mutual coupling model and the impedance phase of the read antenna terminal variation with *Q* factor of LC resonant sensor under high temperature conditions. Design and implemention of the phase-shift detection circuit to detect the resonant frequency of a LC sensor at a distance is described. The circuit outputs a DC voltage signal by comparing phase difference between the sweep-frequency driving signal of the system source terminal and the sweep-frequency signal modulated from the mutual coupling. A pressure test platform under different temperature environments is also achieved. The pressure sensor is placed in a high-temperature environment, while the read antenna is placed in a safe environment protected by the insulating structure. Performances of the pressure sensor are adequately measured by the measurement system, including pressure sensitivity at room temperature, zero drift from room temperature to 800 °C and the pressure sensitivity in the 800 °C high temperature environment. In this paper, the sensor we designed has a zero resonance frequency under 100 KPa, please keep consistent throughout the paper atmospheric pressure, while there will be a frequency drift as the temperature changes, so it is significative and necessary to test how the zero resonance frequency varies with the temperature under 100 KPa atmospheric pressure.

## 2. Analysis of the Telemetric Model

**Figure 1.**
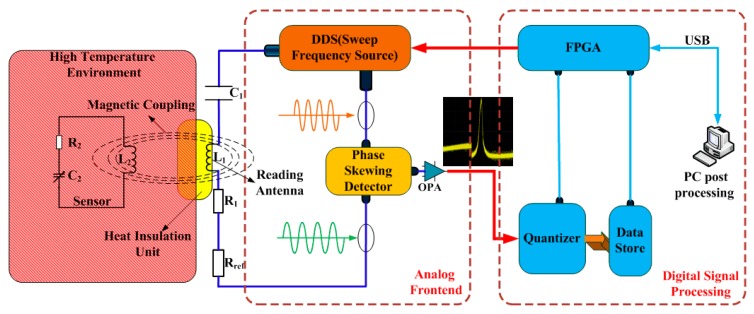
Block diagram of the wireless measurement system.

Due to characteristics of wireless passive sensors without leads and power to transmit signals, a wireless passive test system is designed, as shown in Figure 1. The pressure sensor is placed in the high temperature environment, as shown on the left of Figure 1.

The thermal insulation structure shown in the middle of Figure 1 is made from the mullite material with low thermal conductivity, which ensures the read antenna and the read unit are working in a safe environment. The read unit loading on the terminal of the antenna is configured as shown on the right of Figure 1. Therefore, the entire system can read the shifting of the resonant frequency of wireless passive LC resonant sensor operating under a high-temperature environment to achieve the pressure measurement

### 2.1. The Phase-Shift Readout Method

**Figure 2.**
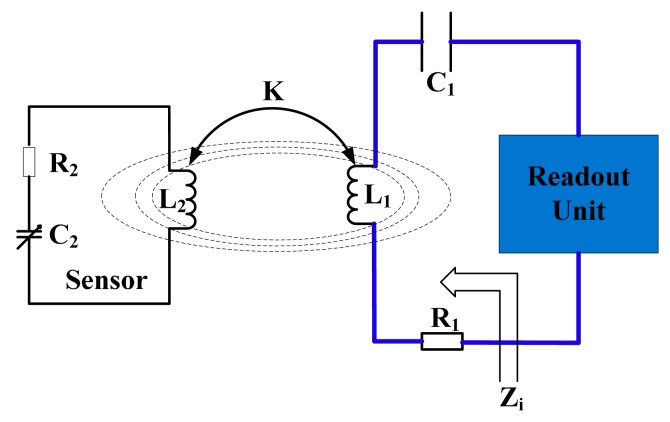
Schematic view of a wireless inductance coupling sensor system.

The variable resonance frequency of an LC sensor is usually characterized by reading the frequency values corresponding to some particular points on the frequency response curve of impedance magnitude or phase on the read antenna terminal. Reference [19] gives the expression of equivalent impedance on the read antenna terminal. As shown in Figure 1, the resonant frequency of sensor can be detected by measuring the various phases and amplitudes of the input impedance which is a function of frequency due to the electromagnetic mutual inductance coupling in a wireless passive sensor measurement system consisted of a LC resonator sensitive device and an inductance reader antenna. The equivalent circuit model of the mutual coupling is given in Figure 2.

The pressure sensor mainly contains a LC resonant circuit, including an inductor *L*_2_, a series resistor *R*_2_ and a variable capacitor *C*_2_. The resonance frequency *f*_0_ of the sensor can be expressed as:

(1) $$f_{0}=\frac{1}{2\pi }\sqrt{\frac{1}{L_{2}C_{2}}-\frac{{R_{2}}^{2}}{{L_{2}}^{2}}}≅\frac{1}{2\pi \sqrt{L_{2}C_{2}}},if\;{R_{2}}^{2}\ll \frac{L_{2}}{C_{2}}$$

The input impedance *Z*_i_ is the total impedance viewed from the read antenna, and according to transformer inductance circuit model, it can be expressed by Equation (2) when *L_1_C_1_ = 1/(4π^2^f_1_^2^)*, *L_2_C_2_ = 1/(4π^2^f_o_^2^)*, *k^2^ = M^2^/(L_1_ × L_2_) and R_2_ = 2πf_o_L_2_/Q.* where *f* is the excitation frequency of the readout unit, M is the coefficient of mutual induction. *k* is the coupling factor between the reader antenna coil and the sensor coil, *L*_1_ is the inductor of the reader antenna inductive coil, *Q* is quality factor of the sensor, *f*_1_ is the self-resonant frequency of the reader antenna. The equivalent input impedance *Z*_i_ can be split up into a real part Equation (4) and an imaginary part Equation (5):

(2) $$Z_{i}=j2\pi fL_{1}\left(1-{\left(\frac{f_{1}}{f_{0}}\right)}^{2}+\frac{k^{2}{\left(\frac{f}{f_{0}}\right)}^{2}}{1-\frac{j}{Q}\left(\frac{f}{f_{0}}\right)-{\left(\frac{f}{f_{0}}\right)}^{2}}\right)$$

(3) $$Q=R_{2}^{-1}{\left(L_{2}C_{2}^{-1}\right)}^{1/2}$$

(4) Re{Zi}=2πfL1k2Qff01+Q2(ff0−f0f)2

(5) Im{Zi}=2πfL1(1−(f1f)2+k2Q21−(ff0)21+Q2(ff0−f0f)2)

With Equations (3) and (4), the phase ∠*Z_i_* of the input impedance is given by:

(6) ∠Zi=arctanIm{Zi}Re{Zi}

By defining Ω=*f* / *f*_0_ = *w*/ *w*_0_, Equation (5) can be rewritten as:

(7) $$∠Z_{i}=\arctan\left[\frac{\left(1-\Omega^{2})+\frac{\Omega^{2}}{Q^{2}}+k^{2}\Omega^{2}(1-\Omega^{2}\right)}{\frac{k^{2}\Omega^{3}}{Q}}\right]$$

(8) $$\left|Z_{i}\right|=\sqrt{{\left[\frac{L_{1}\omega k^{2}\Omega^{3}}{Q\left({\left(1-\Omega^{2}\right)}^{2}+\frac{\Omega^{2}}{Q^{2}}\right)}\right]}^{2}+{\left[L_{1}\omega \left(1+\frac{k^{2}\Omega^{2}\left(1-\Omega^{2}\right)}{{\left(1-\Omega^{2}\right)}^{2}+\frac{\Omega^{2}}{Q^{2}}}\right)\right]}^{2}}$$

**Figure 3.**
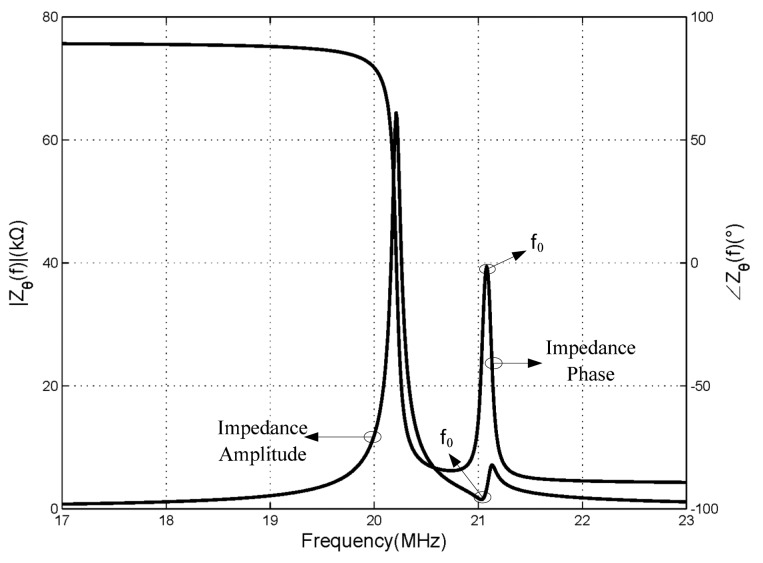
Magnitude and phase of input impedance for a LC reading-antenna.

According to this, as shown in Figure 3, we can extract the resonant frequency of the sensor through the changes of phase extreme value characteristic displayed in the phase-frequency chart. In the reader antenna terminal, the phase of the input impedance characteristics will change from −90° to +90° when there is no mutual coupling before being coupled by the sensor. However, due to the mutual coupling, a sudden change in the shape of the phase response curve occurs. As can be seen from Figure 3, the first curve is the frequency response curve of impedance amplitude, the second curve is the frequency response curve of impedance phase. Due to the mutual inductance coupling, an extreme value point appeared on the impedance amplitude and phase curves. And it is also illustrated in the article [11,16] that the shifting of the frequency of the two points from the amplitude and phase are caused by the variable capacitance of the sensing element.

Coupling factor *k* is the other vital characterization of mutual inductance coupling; *k* is related to geometry size (side length is a), coupling distance (d), metal magnetic permeability (μ_0_) of inductance coil. Under the identical coupling distance, the coupling factor *k* increased as the geometry size a increased. While under the same geometry size a, coupling factor *k* decreased as the coupling distance d increased. In high-temperature environment measurement research, the selected material of the inductance coil is metallic silver paste, whose permeability μ_0_ is almost stable at the temperature ranges from room temperature to 800 °C, while, there is almost no change of geometry size. Therefore, there is no obvious change of the coupling factor *k* in case of the same coupling distance. In the measurement system of this paper, we choose half of length of side, which is a/2, as the coupling distance, so we can obtain the best coupling effect.

### 2.2. The Measurement Method Used in High Temperature

By defining Ω = *f*/*f*_0_ = ω/ω_0_ = 1, Equations (7) and (8) can be simplified as:

(9) $${\left[\left|Z_{i}\right|\right]}_{\begin{array}{l} f=f_{0}\\ \Omega =1 \end{array}}=\sqrt{4\pi^{2}{f_{0}}^{2}{L_{1}}^{2}k^{4}Q^{2}+4\pi^{2}{f_{0}}^{2}{L_{1}}^{2}}$$

(10) $${\left[∠Z_{i}\right]}_{\begin{array}{c} f=f_{0}\\ \Omega =1 \end{array}}=\arctan\frac{1}{k^{2}Q}$$

If the *Q* value of the remote sensor changes to a low level, the peak of the feature points will disappear so that the resonant frequency of the sensor cannot be detected. Therefore, it is meaningful to study the variation of the amplitude and phase of input impedance according to the changes of *Q* factor:

(11) $${\left\{\frac{\partial \left|Z_{i}\right|}{\partial Q}\right\}}_{\begin{array}{l} f=f_{0}\\ \Omega =1 \end{array}}=\frac{4\pi^{2}{f_{0}}^{2}{L_{1}}^{2}k^{4}}{\sqrt{4\pi^{2}{f_{0}}^{2}{L_{1}}^{2}k^{4}+4\pi^{2}{f_{0}}^{2}{L_{1}}^{2}Q^{-2}}}$$

(12) $${\left\{\frac{\partial ∠Z_{i}}{\partial Q}\right\}}_{\begin{array}{l} f=f_{0}\\ \Omega =1 \end{array}}=-\left(\frac{k^{2}}{1+k^{4}Q^{2}}\right)$$

As can be seen from Equations (11) and (12), the change rates of amplitude and phase at the peak are not the same as the *Q* factor changes. In other words, the phase is not more sensitive to the temperature-dependent *Q* factor when compared with the amplitude.

**Figure 4.**
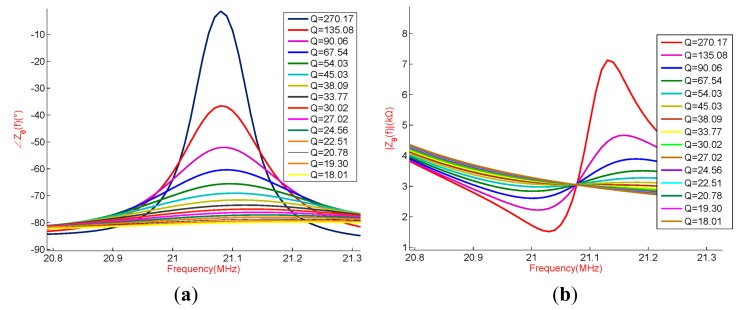
(**a**) Antenna coil phase of input impedance for the quality factor variation; (**b**) Antenna coil Magnitude of input impedance for the quality factor variation.

As can be seen from the Figure 4, when the *Q* factor of the sensor drops to a certain value, the mutative characteristics of the input impedance on the read antenna terminal will disappear so that the resonant frequency of the sensor cannot be read. There are almost no resonance characteristics of impedance magnitude when *Q* factor of the sensor decreases from 270.15 to 45.03, while there are obvious resonance characteristics of the impedance phase when the *Q* factor of the sensor decreases from 270.15 to 27.02. Therefore, our research selects the frequency shifting read method according to impedance phase to measure the resonant frequency of wireless passive LC resonant sensor operating under high-temperature environment.

## 3. Description and Design of the Proposed Measurement System

### 3.1. Conditioning Electronics

**Figure 5.**
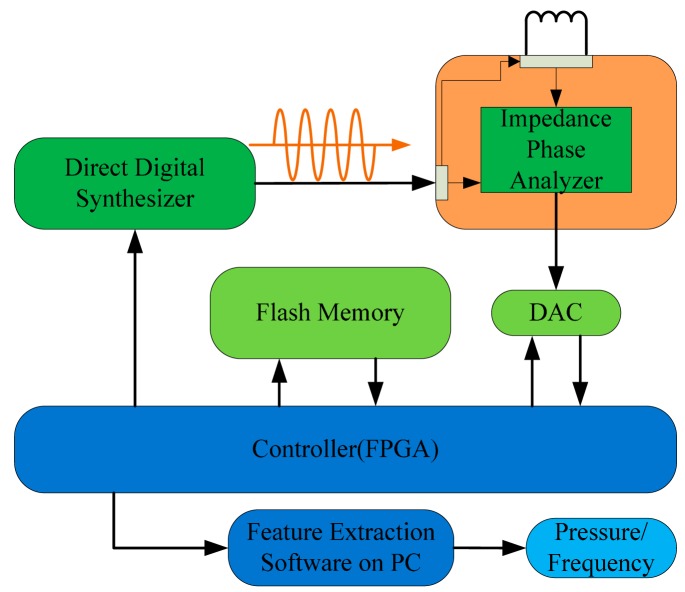
Block diagram of the conditioning electronics.

**Figure 6.**
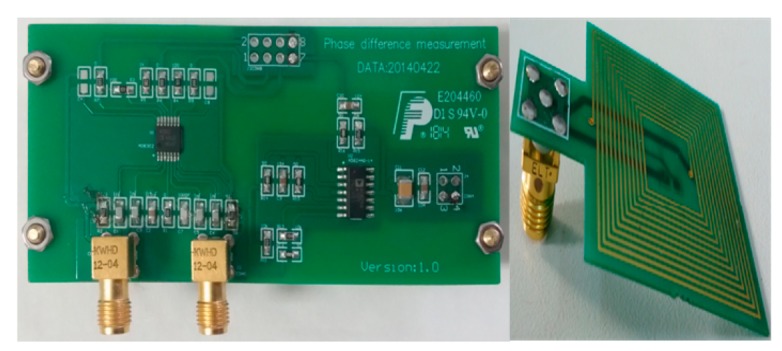
The prototype of the analogy fronted circuit and reading antenna for phase difference detector.

The phase interrogation electronics designed in the readout unit contain three components in total as shown in Figures 5 and 6: the sweep frequency signal synthesizer using direct digital frequency synthesis (DDS) technology, an impedance phase shift analyzer and a FPGA controller as the control unit. The synthesizer generates the linear sweep source which the system requires. The impedance phase shift analyzer detects the phase difference between the signal via the readout antenna and the source signal, then convertes it to a DC signal. The controller generates the working temporal logic of the synthesizer, analog to digital converter (ADC) and flash store. Finally, the measurement data of the quantified phase difference signal is sent to the PC through a USB port for extracting the resonance frequency characteristics.

The sweep frequency signal source synthesizer includes a commercial AD9858 with a working frequency up to 1 GHz and an external input reference clock up to 2 GHz. The AD9858 has 10 internally integrated A/D converters, the frequency resolution (frequency accumulator digits) is 32 bits and it can output a signal up to 400 MHz. In this work, the synthesizer generates a linear sweep signal from 1 to 100 MHz with a chosen 500 MHz reference clock. The output of the synthesizer is amplified by a power amplifier in order to drive the mutual inductance coupling and the signal transmission on the RF cable.

The impedance phase shift analyzer, commercialized by AD8302, mainly contains two broadband logarithmic detectors with precise matching, a phase detector, the output amplifier group, the offset unit and a reference voltage output buffer. It can detect the amplitude ratio and phase difference between the two input signals from low frequency to 2.7 GHz. The analyzer extracts the phase difference information between the source signal generated by synthesizer and the signal coupled with LC resonator remotely through the readout antenna, then converts the −90°–+90° phase difference to a 0–1.8 V DC voltage. After quantification, the data is transferred to the computer for resonance feature extraction by software.

The system controller chooses Field-Programmable Gate Array (FPGA) as the central logic control unit in order to realize the digital control for the frequency sweep source synthesizer, the logic acquisition sequence of ADC and the storage or transmission of the data. The readout unit sends the quantized phase characteristic data to PC for display and processing, meanwhile, stores them in a flash memory unit. The PC analyzes the phase extreme values of the remote LC resonator, and extracts the resonant frequency information.

### 3.2. Sensor Design and Fabrication

**Figure 7.**
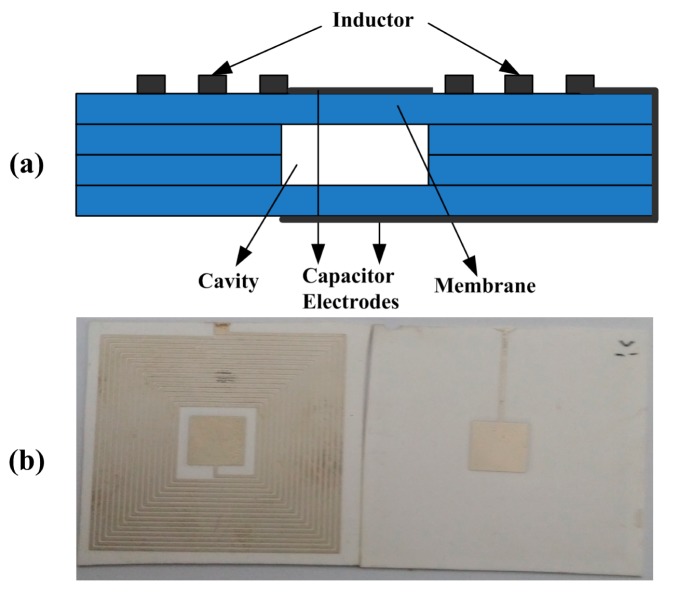
(**a**) Sectional view of the sensor based on alumina ceramic substrate; (**b**) Sensor sample.

Making use of 96% alumina ceramic green tape and Dupont Ag 6142D paste material, a wireless pressure sensor with a pressure deformable square cavity and a planar spiral inductor is designed and fabricated, as shown in Figure 7. The substrate of the sensor is fabricated by four sheets of green tape, the middle two sheets of which are cut to achieve the ceramic cavity holes, and the conductive paste is applied on the ceramic substrate to form a LC resonant circuit.

In high temperature co-fired ceramics production process, the 96% alumina ceramic green tape is sintered at 1500 °C, however, the Ag paste material is sintered at 850 °C. In order to get a complete LC circuit based on the alumina ceramic, we adopted a novel technological process to replace the traditional one in which the alumina ceramic green tapes and Ag paste cannot be co-fired at the same time.

After cutting the alumina ceramic cast film, punching the cavity, stacking and laminating the three manufactured layers with the carbon film, the alumina ceramic green tape is sintered in the sintering furnace at a peak temperature of 1500 °C with a total firing time of 17 h. Then, with the screen printing technology, the four-layer alumina ceramic structure is screened with the Ag paste to form the planar spiral inductor and the capacitance plates. Finally, the alumina ceramic with Ag paste is sintered in a seven-zone belt furnace at 850 °C for 15 min. The complete device is demonstrated in Figure 7b. In terms of the sensor design introduced above, the relevant theoretical geometrical structure parameters of the capacitor and the inductor are given in Table 1. The initial capacitance can be concluded by:

(13) $$C_{0}=\varepsilon_{0}\frac{a^{2}}{t_{g}+\frac{t_{m}}{\varepsilon_{r}}}$$

where *ε*_0_ is the vacuum dielectric constant, *ε_r_* is the relative dielectric constant of the alumina ceramic, *t_m_* is the thickness of total sensitive membranes and *t_g_* is the height of the ceramic cavity.

The initial inductance is:

(14) $$L_{s}=K_{1}\mu_{0}\frac{n^{2}d_{\mathrm{avg}}}{1+K_{2}\rho }$$

where:

(15) $$d_{\mathrm{avg}}=\frac{d_{in}+d_{\text{out}}}{2}$$

(16) $$\rho =\frac{d_{\text{out}}-d_{in}}{d_{\text{out}}+d_{in}}$$

*n* is the number of coil turns, *d_avg_* is the average diameter of coils, *ρ* is the filling ratio, *d_in_* is the inner diameter of inductance coils, *d_out_* is the outer diameter of inductance coils, *μ*_0_ = 4*π* × 10^−7^ and *K*_1_, *K*_2_ is the correlation coefficient, *K*_1_ = 2.34, *K*_2_ = 2.75.

## 4. Experimental Apparatus and Results

**Figure 8.**
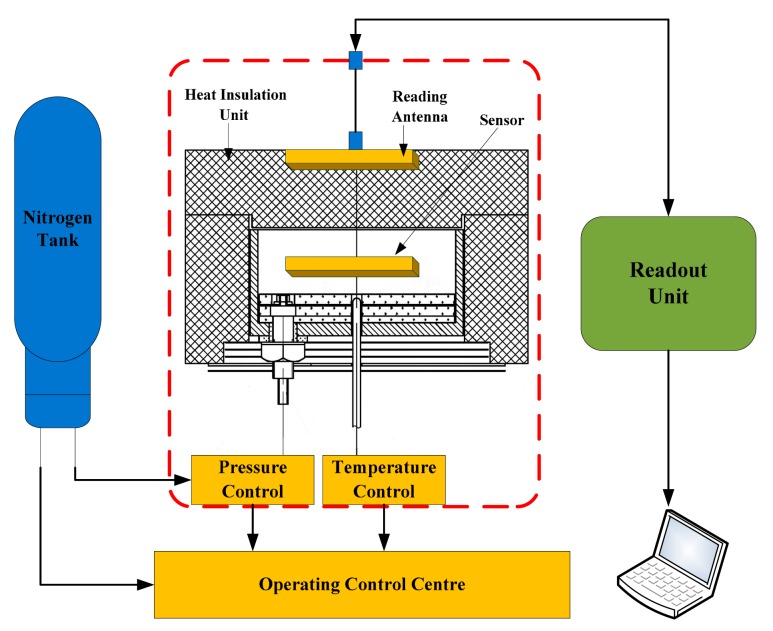
The customized temperature-pressure measurement system using phase readout unit.

A composite high temperature-pressure measurement system, as shown in Figure 8, is designed on the basis of the phase readout unit to achieved 10 bar (the maximum) atmospheric pressure in the temperature range of 1000 °C.

**Figure 9.**
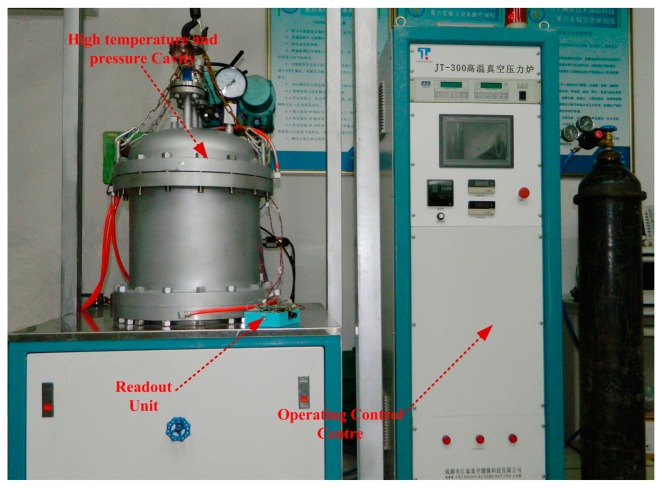
The integrated wireless and passive temperature-pressure test platform.

Measurements are achieved by the system including the pressure measurement from 0 bar to 2 bar at room temperature, the temperature measurement from room temperature to 800 °C with no pressure, and the pressure measurement from 70 KPa to 190 KPa at 800 °C. The experimental setup is shown in Figure 9. The measurement system is composed of a readout unit, a read antenna, a sensor, and a composite high temperature-pressure platform, where temperature is generated by a molybdenum heater and pressure is generated by a nitrogen source and the gas pressure controller.

**Figure 10.**
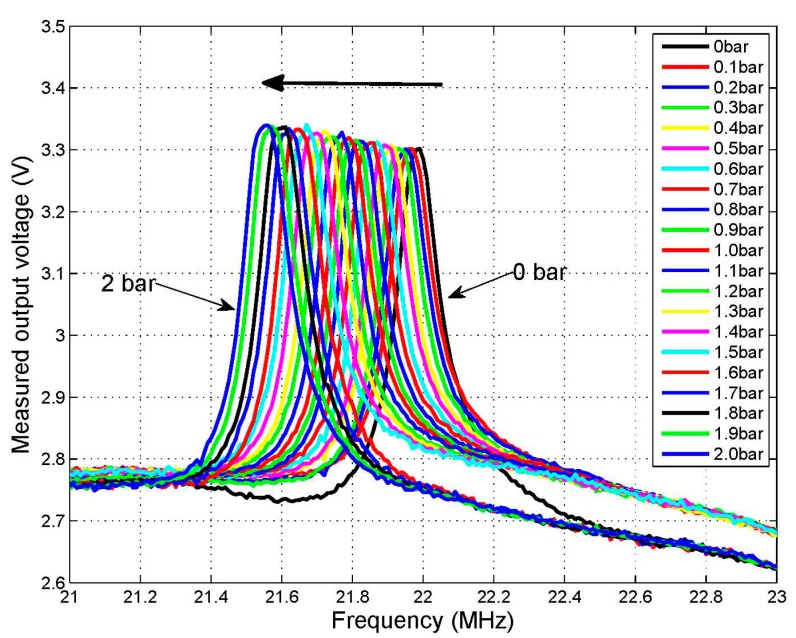
The measured output voltage *versus* frequency from 0 bar to 2 bar.

The verification experiment of the readout unit and sensor has three parts: the first part is the pressure measurement in a room temperature environment; the result is shown in Figure 10. The test data are respectively extracted by the readout apparatus and impedance analyzer; the purpose is to verify the sensor sensitivity performance and that the readout circuit works correctly. The second part is high temperature performance. The third part is pressure sensitive performance in a high-temperature and pressure environment. The test data of parts two and three are extracted all by the phase characteristics reading devices presented in this paper.

**Figure 11.**
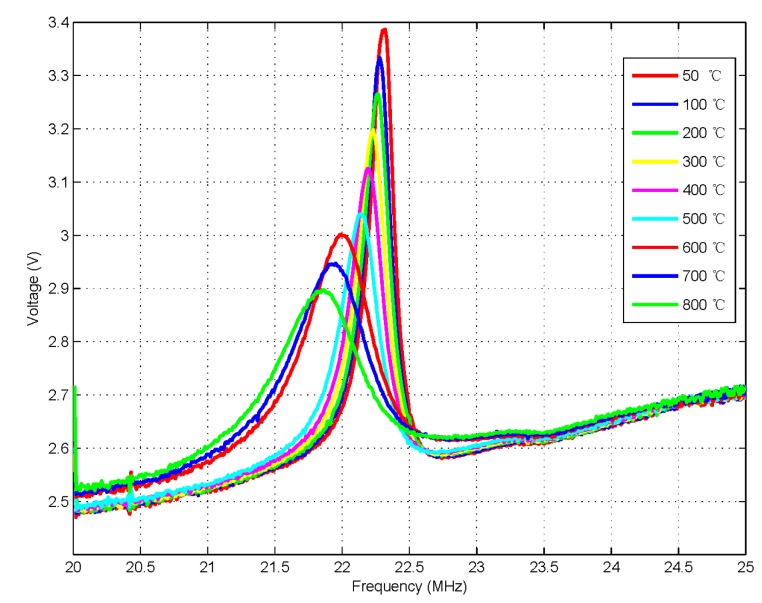
The measured output voltage *versus* frequency from 50 °C to 800 °C.

**Figure 12.**
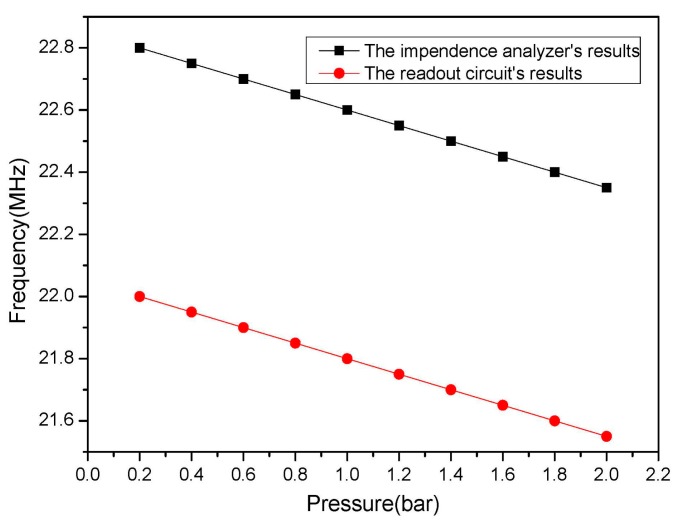
The resonant frequency of the sensor *versus* pressure.

The impedance phase shift analyzer in the readout unit converted the phase difference −90°–+90° to DC voltage 0–1.8 V, and at the same time, the switching voltage is biased in the reading device and the offset value is 2.5 V. The curves in Figures 10 and 11 are plotted by the DC data of the phase characteristics which are obtained from the phase read unit. Figure 10 shows the varying pressure value at room temperature and Figure 11 shows the varying frequency value at high-temperature. Therefore, the voltages in Figures 10 and 12 represent the phase value, and the curves are plotted using the DC voltage data which are extracted through the reading device. The DC curve amplitude of phase characteristics is 2.76–3.32 V in Figure 10, and after subtracting 2.5 V, the bias voltage is 0.26–0.82 V, so the phase change is −26°–−82°. Similarly, the phase characteristics DC curve amplitude is 2.51–3.38 V in Figure 11, so the bias voltage is 0.01–0.88 V after subtracting 2.5 V, therefore the phase change is −1°–−88°.

The experimental results are shown in Figure 10, when the the pressure value varies at room temperature. The test pressure range is 0–2 bar, the sensor frequency variation is 450 KHz, the minimum step of the input pressure is 20 KPa, and the minimum frequency variation is 50 KHz. The curves in Figure 12 are plotted by extracting the extrema of the pressure curves in Figure 10, that is the resonance frequency of the sensor is extracted from the test result which is obtained from the phase read unit. The intercepts of the curves are not the same as the one measured by an impedance analyzer, but the slope is the same, as shown in Figure 12, due to the different distances between the sensor and a reader antenna in the two different testing processes, which verifies the accuracy of the readout unit.

**Figure 13.**
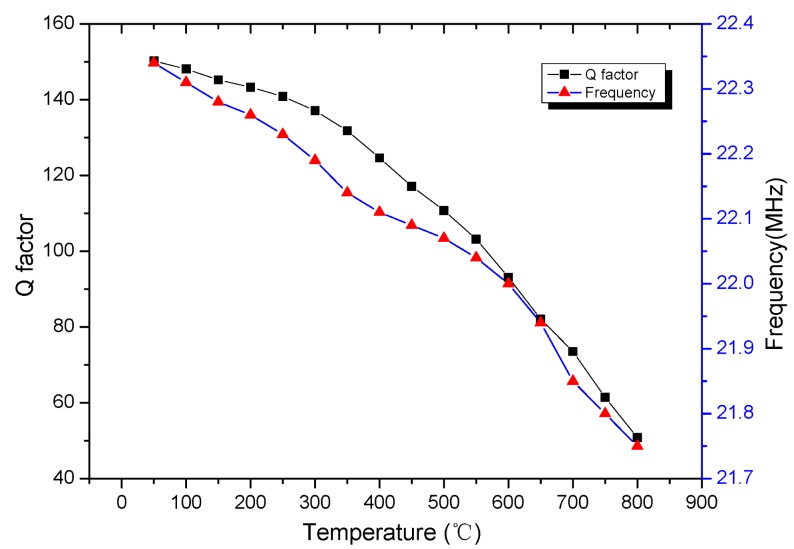
The resonant frequency of the sensor *versus* temperature.

The experimental results are shown in Figures 11 and 13. The curves in Figure 11 plot the DC data of the phase characteristics which is obtained from the phase read unit. Meanwhile, the curves of Figure 13 plot the resonance frequency points which are extracted from the maximum value of the test curves through the software on the computer while varying the temperature value from room temperature to 800 °C with no pressure. As the temperature increases, the resonant frequency and curve peaks change, and the *Q* factor of the sensor decreases from 162.3 to 53.7. Therefore, the pressure sensor is sensitive to temperature, and it also very necessary to take some time to ensure thermal balance before conducting the next static pressure measurement.

As we can see from the phase frequency response curves shown in Figure 11, each point of the curve contains two coordinates, one is the frequency and the other is the phase value. The sensor's mentioned *Q* value is calculated from the peak values in the phase frequency response data curve. Note that in Figure 11, the phase at peak points is 88.6° at the temperature of 50 °C and turns to 39.7° when the temperature rises to 800 °C with a change interval of 48.9°. According to Equation (10) *f* = *f_0_* at peak points, since the coupling coefficient *k* is related to the geometry size and metal permeability of inductance coil, *k* = 0.095 by measurements and it's assumed that the value remains the same in the temperature range. Therefore, *Q* = 150.25 when the generated ${\left[∠Z_{i}\right]}_{\begin{array}{c} f=f_{0}\\ \Omega =1 \end{array}}=88.6{}^{\circ}$ is substituted into Equation (10) at 50 °C, and *Q* = 50.82 when ${\left[∠Z_{i}\right]}_{\begin{array}{c} f=f_{0}\\ \Omega =1 \end{array}}=39.7{}^{\circ}$ at 800 °C which is smaller than the value calculated by the former LCR method, the resonant frequency of the sensor *versus* Q and temperature as shown in Figure 13.

(17) $${\left[∠Z_{i}\right]}_{\begin{array}{l} f=f_{0}\\ \Omega =1 \end{array}}=\arctan\frac{1}{k^{2}Q}$$

**Figure 14.**
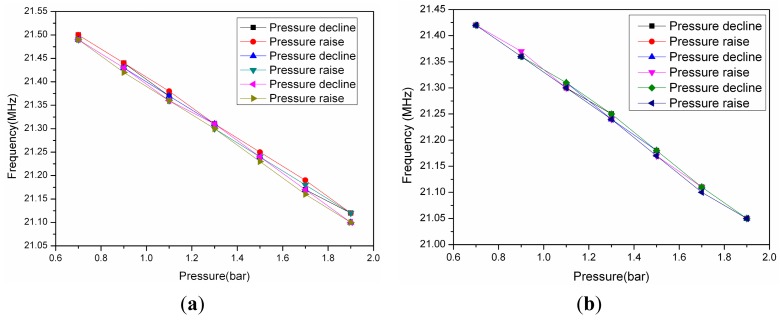
(**a**) The resonant frequency of the sensor *versus* pressure at 800 °C; (**b**) The resonant frequency of the sensor *versus* pressure at 600 °C.

**Figure 15.**
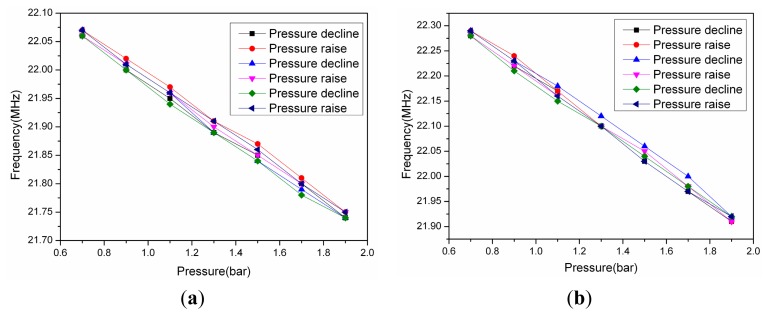
(**a**) The resonant frequency of the sensor *versus* pressure at 400 °C; (**b**) The resonant frequency of the sensor *versus* pressure at 200 °C.

The experimental results are shown in Figure 14. The acquisition method of the curves in Figure 14 is similar to Figure 11. varying the pressure value from 70 KPa to 190 KPa at 800 °C (due to the limitations of the heating element material of composite test platform, we must ensure that the heating element will not be damaged when the test chamber is in an atmospheric environment, so the pressure test range is 70 KPa to 190 KPa rather than 0 KPa to 190 KPa). Before the experiment, the temperature of the composite test platform rises to 800 °C, then after 90 min of heat preservation, when the system reaches a thermal balance, the resonant frequency of the sensor will not change and remains at a certain value. At this point, the container is filled with nitrogen. The variation of the resonance frequency is about 436 KHz, and the minimum stepping value is 20 KPa. The results are shown in Figures 14 and 15, from which it can be calculated that the linearity of the sensor is 0.93%, 1.53%, 1.49%, 0.68%, the repeatability is 6.6%, 2.77%, 5.98%, 7.93%, the hysteretic error is 1.67%, 0.9%, 2.53%, 2.19%, and the sensor sensitivity is 374 KHz/bar, when the system reaches thermal equilibrium at 800 °C, 600 °C, 400°C, 200°C, respectively.

## 5. Conclusions

In this paper, we present a mutual coupling electromagnetic model for high-temperature applications, analyze how the impedance on the antenna terminal varied with the *Q* factor of the LC resonant sensor, and design a wireless passive pressure measurement system for high-temperature environments, which consists of a ceramic-based LC resonant sensor, a readout device for impedance difference, heat insulating material, and a composite temperature-pressure test platform. We perform measurements on the pressure sensor using the system, including pressure measurements from 0 bar to 2 bar at room temperature, temperature measurements from room temperature to 800 °C with no pressure, and pressure measurements from 70 KPa to 190 KPa at 800 °C. The results show that the linearity of the sensor is 0.93%, the repeatability is 6.6%, the hysteretic error is 1.67%, and the sensor sensitivity is 374 KHz/bar. In our future research, we will complete the dynamic performance measurements in a higher temperature environment by improving the performance of the sensor and measurement system.

## Figures and Tables

**Table 1.** Design parameters of the capacitor and inductor.

**Parameter**	**Values**
Side length of the square electrode (mm)	8
Cavity height (μm)	∼80
Thickness of one sensitive membrane (μm)	∼80
Width/Spacing (mm)	0.4/0.4
Inner diameter (mm)	12
Outer diameter (mm)	34.8
NO. of turns	16
The initial inductance (*μH*)	7.53
The initial capacitance (*pF*)	5.64
The initial resonance frequency (*MHz*)	24.42
